# Association of Trimethylamine, Trimethylamine N-oxide, and Dimethylamine with Cardiovascular Risk in Children with Chronic Kidney Disease

**DOI:** 10.3390/jcm9020336

**Published:** 2020-01-25

**Authors:** Chien-Ning Hsu, Guo-Ping Chang-Chien, Sufan Lin, Chih-Yao Hou, Pei-Chen Lu, You-Lin Tain

**Affiliations:** 1 Department of Pharmacy, Kaohsiung Chang Gung Memorial Hospital, Kaohsiung 833, Taiwan; chien_ning_hsu@hotmail.com; 2 School of Pharmacy, Kaohsiung Medical University, Kaohsiung 807, Taiwan; 3 Center for Environmental Toxin and Emerging-Contaminant Research, Cheng Shiu University, Kaohsiung 833, Taiwan; guoping@csu.edu.tw (G.-P.C.-C.); linsufan2003@gmail.com (S.L.); 4 Super Micro Mass Research and Technology Center, Cheng Shiu University, Kaohsiung 833, Taiwan; 5 Department of Seafood Science, National Kaohsiung University of Science and Technology, Kaohsiung 811, Taiwan; chihyaohou@gmail.com; 6 Department of Pediatrics, Kaohsiung Chang Gung Memorial Hospital and College of Medicine, Chang Gung University, Kaohsiung 833, Taiwan; alexiellu@gmail.com; 7 Institute for Translational Research in Biomedicine, Kaohsiung Chang Gung Memorial Hospital and Chang Gung University, College of Medicine, Kaohsiung 833, Taiwan

**Keywords:** ambulatory blood pressure monitoring, cardiovascular disease, children, chronic kidney disease, dimethylamine, gut microbiota, hypertension, trimethylamine, trimethylamine N-oxide

## Abstract

Chronic kidney disease (CKD) is associated with high risk for cardiovascular disease (CVD). Gut microbiota-dependent metabolites trimethylamine (TMA), trimethylamine N-oxide (TMAO), and dimethylamine (DMA) have been linked to CKD and CVD. We examined whether these methylamines are correlated with cardiovascular risk in CKD children. A total of 115 children and adolescents with CKD stage G1–G4 were enrolled in this cross-sectional study. Children with CKD stage G2–G4 had higher plasma levels of DMA, TMA, and TMAO, but lower urinary levels of DMA and TMAO than those with CKD stage G1. Up to 53% of CKD children and adolescents had blood pressure (BP) abnormalities on 24-h ambulatory BP monitoring (ABPM). Plasma TMA and DMA levels inversely associated with high BP load as well as estimated glomerular filtration rate (eGFR). Additionally, CKD children with an abnormal ABPM profile had decreased abundance of phylum *Cyanobacteria,* genera *Subdoligranulum*, *Faecalibacterium,* *Ruminococcus,* and *Akkermansia*. TMA and DMA are superior to TMAO when related to high BP load and other CV risk factors in children and adolescents with early-stage CKD. Our findings highlight that gut microbiota-dependent methylamines are related to BP abnormalities and CV risk in pediatric CKD. Further studies should determine whether these microbial markers can identify children at risk for CKD progression.

## 1. Introduction

Chronic kidney disease (CKD) is associated with high risk for cardiovascular disease (CVD), not just for adults but also for children [1,2]. Even though overt CVD barely presents in children, the process of atherosclerosis can originate in early life. Therefore, heightened efforts are needed to identify CKD children at higher risk for CVD during their lifetimes to develop effective interventions for preemption [3]. Several noninvasive procedures, such as 24-h ambulatory blood pressure monitoring (ABPM) [4], ambulatory arterial stiffness index (AASI) [5], and left ventricular mass [6], are available to evaluate risk for CVD in children with CKD.

Gut microbiome has been identified as a source of pathogenic mediators in CKD [7]. Certain gut microbiota-derived metabolites have been shown to correlate with CVD in patients with CKD [8,9]. Among them, trimethylamine N-oxide (TMAO) has attracted increasing notoriety recently as a causative factor in various CVD. Emerging evidence has linked higher blood levels of TMAO with a higher risk of CVD [10].

TMAO production is a two-step process. The first step involves the liberation of trimethylamine (TMA) from dietary precursors (e.g., choline and carnitine) by gut microbes. In the second step, TMA is oxidized to TMAO by hepatic flavin-containing monooxygenases (FMOs). Both TMAO and TMA can be subsequently converted to dimethylamine (DMA). Thus, the plasma TMAO-to-TMA ratio may reflect FMO activity, while DMA-to-TMAO ratio is related to TMAO-metabolizing activity.

Although plasma levels of TMAO, TMA, and DMA are all increased in uremic patients [11], little attention has been paid to understand the role of their levels and combined ratios in the development of CVD in children with early-stage CKD. Because TMAO, TMA, and DMA are all tightly linked to each other and because all these methylamines are excreted into urine [12], we assume that simultaneous analysis of their combined ratios in the plasma and urine may provide more information to reflect the TMA–TMAO metabolic pathway. Thus, we assessed the association between CVD assessments, microbial markers, and TMA–TMAO metabolic pathway in early pediatric CKD.

## 2. Materials and Methods

### 2.1. Study Population

This is a cross-sectional study of 115 children and adolescents with CKD who were enrolled in the Kaohsiung Chang Gung Memorial Hospital from January 2019 to December 2019. Human subjects review board approval was obtained from the Institution Review Board and Ethics Committee of Chang Gung Medical Foundation, Taoyuan, Taiwan (Permit number: 201701735A3C501). Our study protocol was conducted in accordance with the 1964 Helsinki Declaration and its later amendments. Written informed consent was obtained from all participants. CKD is defined as abnormal kidney structure or function lasting more than three months [13]. We calculated estimated glomerular filtration rate (eGFR) with the Schwartz formula according to body height and blood creatinine (Cr) level [14]. Kidney damage included proteinuria, urine sediment abnormalities, or structural abnormalities and was detected by histology or imaging. Participants were categorized according to eGFR (mL/min/1.73 m^2^): G1 ≥90, G2 60–89, G3 30–59, or G4 15–29. The causes of kidney diseases were divided to two categories: Congenital anomalies of the kidney and urinary tract (CAKUT) or non-CAKUT. CAKUT structural anomalies ranged from renal agenesis, kidney hypo-/dysplasia, horseshoe kidney, duplex collecting system, multi-cystic kidney dysplasia, posterior urethral valves, and ureter abnormalities [15]. Patients were excluded from the study if they (1) had a history of congenital heart disease; (2) were already documented as being pregnant; (3) had eGFR <15 mL/min/1.73 m^2^ or were on dialysis maintenance, or had ever received renal transplantation; (4) were unable to cooperate with CV assessment.

### 2.2. Biochemical Analysis

Fasting plasma and spot urine samples were stored at −80 °C until analysis. We directed the family to have their children avoid excessive intake of foods rich in choline and carnitine (e.g., eggs, fish, or red meat) for 1 week before blood and urine sampling. Blood urea nitrogen (BUN), creatinine, total cholesterol, low-density lipoprotein (LDL), triglyceride, sodium, potassium, calcium, phosphate, uric acid, glucose, hemoglobin, hematocrit, and urine total protein-to-creatinine ratio were measured by the hospital central laboratory as described previously [16].

### 2.3. Liquid Chromatography–Mass Spectrometry (LC–MS/MS) Analysis

We analyzed plasma and urinary concentrations of DMA, TMA, and TMAO by LC–MS/MS analysis using an Agilent 6410 Series Triple Quadrupole mass spectrometer (Agilent Technologies, Wilmington, DE, USA) equipped with an electrospray ionization source [16]. The multiple-reaction-monitoring mode was set up using characteristic precursor-product ion transitions to detect m/z 46.1→30, m/z 60.1→44.1, and m/z 76.1→58.1, for DMA, TMA, and TMAO, respectively. Separation was performed in the Agilent Technologies 1200 HPLC system consisting of an autosampler and a binary pump. Chromatographic separation was performed on a SeQuant ZIC-HILIC column (150 × 2.1 mm, 5 µm; Merck KGaA, Darmstadt, Germany) protected by an Ascentis C18 column (2 cm × 4 mm, 5 µm; Merck KGaA, Darmstadt, Germany). Diethylamine was added to samples as an internal standard. The mobile phase containing methanol with 15mmol/L ammonium formate (phase A) and acetonitrile (phase B) was used at a ratio of 20:80 (phase A: phase B), with the flow rate set as 0.3–1 mL/min. The urinary concentration of each methylamine was corrected for urine Cr concentration, which was represented in ng/mg Cr.

### 2.4. Blood Pressure Measurement and Echocardiography

Participants were instructed to measure office blood pressure (BP) at the clinic visit after a 5 min rest with at least 1 min between recordings. The mean value was used for calculations. The 24-h ABPM data were collected for subjects aged 6–18 years, handled by an experienced specialist nurse as described previously [16]. We used the Oscar II monitoring device (SunTech Medical, Morrisville, NC) to measure BP and pulse rate at 20-min intervals over 24 h. The participants and their parents were requested to complete a diary of sleeping and waking times, as well as activities that may influence BP measurements. An abnormal ABPM profile was defined as (1) awake, asleep, systolic, or diastolic BP loads ≥95th percentile based on gender and height using ABPM reference data [17]; (2) awake, asleep, systolic or diastolic BP load ≥25%; and (3) asleep decrease of BP load <10% compared with average awake BP load. Next, the ambulatory arterial stiffness index (AASI) is an index derived from 24-h ABPM for the evaluation of arterial stiffness [5]. The AASI was defined as 1 minus the regression slope of diastolic BP (DBP) on systolic BP (SBP) [18]. Echocardiographic examination was performed with commercially available machines (Philips IE33 system, Philips, Bothell, WA, USA). The left ventricular (LV) mass was calculated using images obtained in the parasternal long-axis or short-axis view of the left ventricle by M-mode echocardiography. The LV mass index (LVMI) was obtained by indexing LV mass to height^2.7^ [19].

### 2.5. Analysis of Gut Microbiota Composition

Metagenomic DNA was extracted from frozen fecal samples. Simple centrifugation processing was carried out to remove impurity, proteins, and other organic compounds. As described previously [16], all polymerase chain-reaction amplicons were mixed together with the Biotools Co., Ltd. (Taipei, Taiwan) for sequencing using an Illumina Miseq platform (Illumina, CA, USA). Amplicons were prepared according to the 16S Metagenomics Sequencing Library Preparation protocol (Illumina, CA, USA), and sequenced using the variable V3–V4 regions of the 16S rRNA gene with the Illumina MiSeq platform (Illumina, CA, USA) in paired-end mode with 600-cycle sequencing reagent. The sequences were analyzed using QIIME version 1.9.1. A median of 116,776 raw sequencing reads and 79,067 effective tag sequences per sample was obtained, respectively. Sequences with a distance-based similarity of 97% or greater were grouped into operational taxonomic units (OTUs) using the USEARCH algorithm. The phylogenetic relationships were determined based on a representative sequence alignment using Fast-Tree. Shannon’s index accounting for both abundance and evenness of the taxa present was analyzed by QIIME version 1.9.1. We evaluated the β-diversity changes in gut microbiota across groups by the Partial Least Squares Discriminant Analysis (PLS-DA) and the weighted or unweighted UniFrac distances. To determine the significantly differential taxa, we applied linear discriminant analysis effect size (LEfSe) to compare samples between groups. The LEfSe uses linear discriminant analysis (LDA) to estimate the effect size of each differentially abundant feature. The threshold of the linear discriminant was set to 3.

### 2.6. Statistical Analysis

Continuous variables were expressed with the median (25th–75th percentile), and categorical variables were indicated by number (%). The Mann–Whitney U test or Chi-square test was used to test the differences in variables between children with normal and abnormal ABPM. The associations between variables were examined using Spearman’s correlation coefficient. A linear regression model was performed, followed by the stepwise multivariable analyses integrating relevant parameters to explain BP load, AASI, and LV mass. A value of *p* < 0.05 was considered statistically significant. Analyses were performed using the Statistical Package for the Social Sciences (SPSS) software 14.0 (Chicago, IL, USA).

## 3. Results

Characteristics of the study subjects are shown in Table 1. A total of 115 children and adolescents with CKD were enrolled in this study, including 79 G1 subjects (68.7%), 27 G2 subjects (23.5%), seven G3 subjects (6.1%), and two G4 subjects (1.7%). The median age was 11.3 years. Our study population had a slight preponderance of males (M:F = 1.4:1). The median eGFR was 100.7 mL/min/1.73 m^2^, demonstrating most participants were in the early stage of CKD. The major cause of CKD was due to CAKUT (66.1%). Among them, 49 cases (42.6%) had office BP exceeding the 95th percentile for age, gender, and height. As illustrated in Table 1, in terms of biochemical parameters, most patients were well controlled for common complications of CKD. Thirty-one patients (27%) displayed proteinuria. There was a trend toward moderate hyperlipidemia: 23 (20%) and 10 (9%) patients were above the upper normal limit of 200 and 130 mg/dL for total cholesterol and triglyceride, respectively. Moreover, 20 patients (17%) had hyperuricemia. Neither severe anemia nor hyperkalemia was observed.

Table 2 summarizes the results of cardiovascular assessments in children and adolescents with CKD: 75 patients (65%) aged 6–18 years had undergone 24-h ABPM. In total, 53% (40/75) of them had at least one BP load abnormality. Among them, seven (9%), seven (9%), and eight patients (11%) had SBP or DBP load ≥95th percentile at 24-h, awake, and asleep stages, respectively. Additionally, there were 23 patients (31%) with BP load ≥25% and 31 patients (41%) with a non-dipping nocturnal BP profile. CKD children with stage G2–G4 had a higher awake SBP load than that in CKD stage 1. Moreover, the cases with asleep BP ≥95th percentile and BP load ≥25% were greater in CKD stage G2–G4 vs. stage G1 group. The AASI and LV mass were higher in children with CKD stage G2–G4 than those with stage G1. However, LVMI was not different between the two groups.

We next analyzed methylamines in the plasma and urine. Children with CKD stage G2–G4 had higher plasma levels of DMA, TMA, and TMAO compared to those with CKD stage 1 (Table 3). Conversely, urinary levels of DMA and TMAO were lower in patients with CKD stage G2–G4 vs. stage G1. However, both TMAO-to-TMA and DMA-to-TMAO ratios in the plasma and urine did not differ between the two groups. Additionally, fractional excretion of DMA, TMA, and TMAO was comparable between CKD children with stage G1 and stage G2–G4.

**Figure 1:**
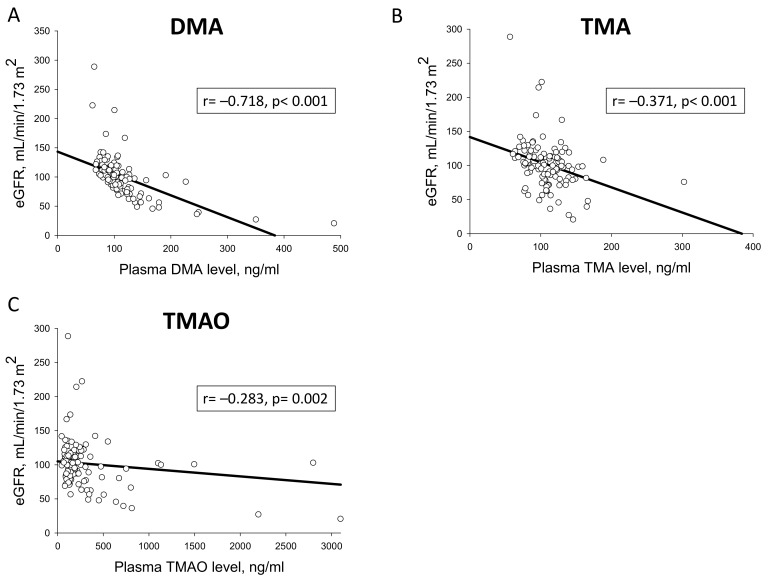
Correlation of plasma (**A**) DMA, (**B**) TMA, and (**C**) TMAO levels (ng/mL) with estimated glomerular filtration rate (eGFR) (ml/min/1.73m^2^) by Spearman’s correlation coefficient. DMA = dimethylamine; TMA = trimethylamine; TMAO = trimethylamine N-oxide.

Using data pooled from all subjects, correlations between plasma and urinary methylamine levels and CV risk factors were analyzed (Table 4). We observed that urinary DMA level was negatively correlated with awake SBP load (*r =* −0.235, *p* = 0.043), asleep SBP load (*r =* −0.289, *p* = 0.012), awake DBP load (*r =* −0.288, *p* = 0.012), and LV mass (*r =* −0.554, *p* < 0.001). Additionally, LV mass exhibited negative correlations with urinary TMA (*r =* −0.226, *p* = 0.016) and TMAO levels (*r =* −0.324, *p* < 0.001). In CKD children and adolescents, there were significantly inverse correlations between eGFR and plasma DMA (*r =* −0.718, *p* < 0.001), TMA (*r =* −0.371, *p* < 0.001), and TMAO (*r =* −0.283, *p* = 0.002) (Figure 1).

As illustrated in Table 5, associations between methylamines and CV risk were further examined in a multivariate linear regression model. To specify the exact role of each methylamine biomarker in CV risk, a multivariate linear regression model using the stepwise selection was applied for age, sex, eGFR, and other methylamines. In the best predictive model (*r =* 0.479, *p* < 0.001), office DBP was associated with urinary TMA (*p* = 0.009) as well as plasma TMA level (*p* = 0.02), controlling for age. Urine DMA-to-TMAO ratio was inversely associated with office DBP controlling for age (*r =* 0.441, *p* < 0.001). A positive association between plasma TMA level and awake DBP was found in the adjusted regression model (*r =* 0.283, *p* = 0.015). Additionally, plasma TMA level was associated with asleep DBP (p = 0.036), controlling for age. We also found that urine DMA-to-TMAO ratio had a negative association with LV mass controlling for age and sex (*r =* 0.771, *p* < 0.001). Furthermore, an inverse association between plasma DMA-to-TMAO ratio and LVMI was found in the adjusted regression model controlling for eGFR and sex (*r =* 0.465, *p* < 0.001).

**Figure 2:**
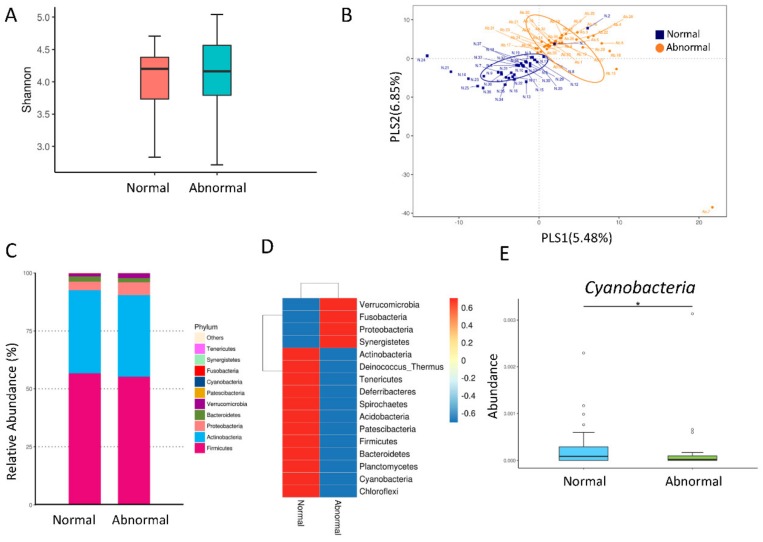
(**A**) Fecal bacterial α-diversity in CKD children with normal and abnormal ABPM represented by the Shannon’s diversity indexes. (**B**) β-diversity changes in gut microbiota between CKD children with normal and abnormal ABPM by the partial least squares discriminant analysis (PLS-DA). (**C**) Relative abundance of top 10 phyla of the gut microbiota between the normal and abnormal ABPM group. (**D**) Heat map of 16S rRNA gene sequencing analysis of gut microbiome at the phylum level. (**E**) The abundance of phylum *Cyanobacteria* in CKD children with normal vs. abnormal ABPM. The asterisk indicates *p* < 0.05.

We further analyzed the composition of the gut microbiota. We determined α-diversity as the mean species richness, quantified by the Shannon index, and found that there was no significant difference between CKD children with normal vs. abnormal ABPM (Figure 2A; *p* = 0.723). The β-diversity was measured by calculating the unweighted UniFrac distances between each pair of samples, and the unweighted UniFrac distance matrix was measured and visualized using a partial least squares discriminant analysis (PLS-DA) analysis. The score plots of PLS-DA analysis showed that two groups were well separated (Figure 2B). The unweighted UniFrac results differed from the normal, and the abnormal ABPM group reached significance (*p* = 0.005). At the phylum level, we observed that the main phyla were *Firmicutes*, *Actinobacteria*, *Proteobacteria*, *Bacteroidetes*, and *Verrucomicrobia* (Figure 2C). Although several phyla showed lower abundance in the abnormal vs. normal ABPM group (Figure 2D), only phylum *Cyanobacteria* reached the significance (*p* < 0.05; Figure 2E). Moreover, the *Firmicutes* to *Bacteroidetes* ratio was comparable between two groups.

**Figure 3:**
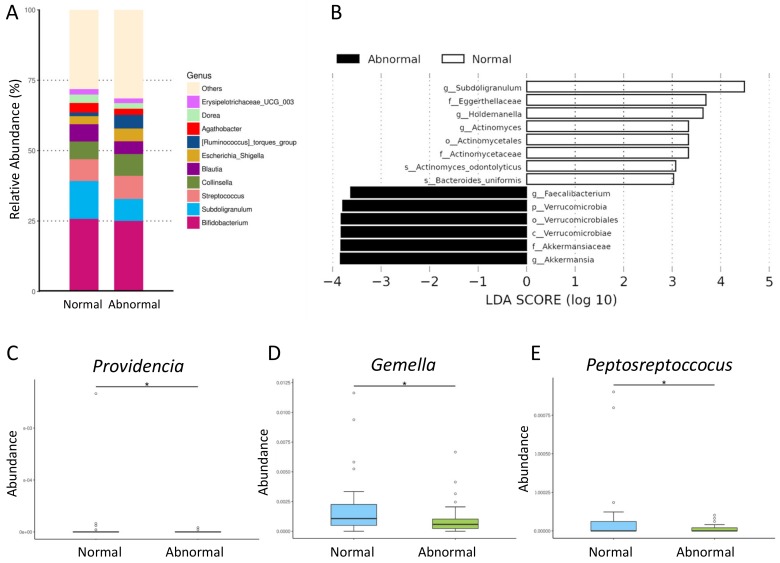
(**A**) Relative abundance of top 10 genera of the gut microbiota in CKD children with normal and abnormal ABPM group. (**B**) Linear discriminant analysis effect size (LEfSe) to identify the taxa that were significantly different between normal vs. abnormal ABPM group. The threshold of the linear discriminant was set to 3. (**C**) The abundance of genus *Providencia*, (**D**) *Gemella*, and (**E**) *Peptosreptoccocus* in CKD children with normal vs. abnormal ABPM. The asterisk indicates *p* < 0.05.

At the genus level (Figure 3A), the abundances of most top 10 genera were not different between the normal and abnormal ABPM group, except genera *Subdoligranulum* (*p* = 0.034) and *Ruminococcus* (*p* = 0.033). Finally, we ran the LEfSe algorithm to identify metagenomic biomarkers (Figure 3B). Our results identified that genera *Subdoligranulum*, *Holdemanella*, and *Actinomyces* are detected by LEfSe with a high LDA score (more than three orders of magnitude), reflecting marked higher abundance in the normal ABPM group. In contrast, the abnormal ABPM group had decreased abundance of genera *Faecalibacterium* and *Akkermansia*. Additionally, the abnormal ABPM group showed a decreased abundance of genera *Providencia* (Figure 3C), *Gemella* (Figure 3D), and *Peptosreptoccocus* (Figure 3E) (all *p* < 0.05).

## 4. Discussion

To the best of our knowledge, this study is the first to evaluate the association between gut microbiota-dependent methylamines and CV risk in early-stage pediatric CKD. The key findings are (1) children with CKD stage G2–G4 had higher plasma levels of DMA, TMA, and TMAO, but lower urinary levels of DMA and TMAO compared to those with CKD stage G1; (2) plasma TMA and DMA levels were not only inversely associated with high BP load, but also eGFR; (3) CKD children with abnormal and normal ABPM associated into two distinct enterotypes; (4) CKD children with an abnormal ABPM profile had decreased abundance of phylum *Cyanobacteria,* genera *Subdoligranulum*, *Faecalibacterium, Ruminococcus,* and *Akkermansia*; and (5) TMA and DMA are superior to TMAO related to CV risk in early-stage CKD children.

In the current study, more than half of CKD children exhibited BP abnormalities on ABPM. Our data showed the cases with nocturnal hypertension and increased BP load were greater in children with CKD stage G2–G4 than those with stage G1. Of note is that up to 48% of children with CKD stage G1 had BP abnormalities on ABPM. Our findings are consistent with previous reports wherein hypertension is extremely prevalent in CKD children, even in an early stage [20,21,22,23]. The present study supported the notion that ABPM is superior to office BP in identifying children with BP abnormalities [24]. As expected, the severity of CKD is associated with certain markers of CV risk, like AASI and LV mass in the current study. AASI, an index of arterial stiffness, has been proposed as a surrogate marker to predict CV morbidity and mortality [18]. Our previous report demonstrated that high AASI is related to BP abnormalities in CKD children, even in an early stage [23]. The present results showed AASI was higher in children with CKD stage G2–G4 than those with stage G1, which ties well with the previous study in that high AASI worsens GFR decline in adult CKD patients [25]. Left ventricular hypertrophy is an index of target organ damage as well as a risk factor for CVD in adult CKD patients [26]. In keeping with previous studies showing that LV mass is greater in CKD children on dialysis and ABPM correlates with LV hypertrophy in CKD children [6,27,28], we observed children with advanced CKD displayed higher degree of LV mass and BP abnormalities.

Gut microbiota-derived metabolites TMAO, TMA, and DMA from dietary methylamines have recently gained much attention due to their high association with CV risk [8,9,10,29,30]. In the present study, our results are consistent with previous reported data in CKD adults [31,32], showing that TMAO is increased in CKD with a weak inverse correlation (*r =* −0.283) between plasma TMAO level and eGFR. Our results are also in agreement with a previous report in CKD adults showing urinary excretion is a dominant route for TMAO elimination with a steady fractional excretion of TMAO, regardless of the CKD stages [32]. However, we observed lower urinary TMAO levels in children with CKD stage G2−G4 than those with stage G1. Either increased or decreased urinary TMAO levels have been reported in adult CKD patients [32,33]. Conflicting results are likewise seen in this study showing that decreased urinary but increased plasma TMAO levels in children with advanced-stage CKD may represent reduced renal excretion of circulating TMAO or increased local renal TMAO metabolism. Although observational and experimental studies suggest a positive correlation between high plasma TMAO levels and increased CV risk [10], data are contradictory and the underlying mechanism is not yet validated [34]. Therefore, it is debated whether TMAO is harmful or beneficial for CV health. Our data showed no significant association between TMAO and most CV surrogate markers, except that urinary TMAO level had a negative correlation with LV mass. Therefore, whether plasma and urinary TMAO may aid in predicting CVD in early-stage pediatric CKD awaits further elucidation.

Strikingly, less attention has been paid to the TMAO precursor TMA in CV risk. TMA is generated by the metabolism of gut microbes using dietary precursors such as choline or carnitine [34]. Like TMAO, TMA is considered as a uremic toxin [29]. Results from this study identified plasma TMA level is increased in CKD children, which is in accordance with data from CKD adults [29,35]. Children with stage G2–G4 had higher plasma TMA levels than those with stage G1. There was also a trend towards a higher plasma TMAO-to-TMA ratio, an index of FMO activity, in this group. Taking into account that plasma TMA and TMAO levels both are simultaneously elevated in children with advanced CKD, our data suggest their increases could be due to increased gut bacterial production and TMA-to-TMAO conversion. Of note, eGFR exhibited a stronger correlation with the TMA than with TMAO. Additionally, TMAO and BP abnormalities were unrelated, whereas plasma TMA level was positively correlated with awake DBP, asleep DBP, and office DBP. Our findings suggest the superiority for the TMA over TMAO as a cardiovascular risk index in children with early-stage CKD.

Another important result of this study is the description of an inverse association between plasma DMA level and cardiovascular risk. Since we found a strong inverse association between plasma DMA level and eGFR, high plasma level but low urinary level of DMA in advanced CKD is possible due to decreased renal excretion. On the other hand, both TMAO and TMA can be metabolized to DMA. It is presumably that an increased plasma DMA level in children with advanced CKD is due to, at least in part, increased gut microbiota-derived TMA and TMAO production. Our previous study showed that urine DMA level was negatively associated with asleep BP load in CKD children [36]. In the present study, we further confirm that urinary DMA level is inversely associated with awake SBP load, asleep SBP load, awake DBP load, and LV mass. These findings suggest that DMA is superior to TMAO in predicting CV risk in early-stage pediatric CKD. On the other hand, our data demonstrated that DMA-to-TMAO ratio, but not TMAO, in the plasma and urine related to several CV risk markers. Given that phases of TMAO synthesis and metabolism happens simultaneously and that DMA-to-TMAO is considered as an index of TMAO-metabolizing activity, data from this study suggest that measures of the combined ratio instead of individual methylamine (i.e., DMA, TMA, and TMAO) may provide us a better understanding of TMA-TMAO metabolic pathway on CVD in CKD children. On the other hand, a previous study demonstrated that the presence of gut microbes might not be essential for DMA production as its excretion is the same in germ-free and control animals [37]. Therefore, DMA might be produced from other endogenous pathways. Indeed, DMA is also a derivative of nitric oxide (NO) metabolism [38]. Asymmetric dimethylarginine (ADMA), an endogenous inhibitor of NO synthase, can be metabolized by dimethylarginine dimethylaminohydrolase to generate DMA and citrulline [38]. Noteworthily, DMA can come from the TMA−TMAO pathway as well the ADMA−NO pathway, and TMAO and ADMA both are uremic toxins related to cardiovascular risk [34,39]. Whether DMA links two pathways together to play a role in the development of hypertension in CKD awaits further elucidation.

In the current study, CKD children with abnormal and normal ABPM associated into two distinct enterotypes, represented by β-diversity changes. Our data support the notion that gut microbiota is linked to the development of hypertension [40,41]. At the phylum level, the abundance of *Cyanobacteria* was lower in the abnormal vs. normal ABPM group. This finding is in line with an animal study showing that minocycline-treated Dahl salt-sensitive rats developed hypertension related to a decrease in *Cyanobacteria* [42]. Although the *Firmicutes*-to-*Bacteroidetes* ratio has been linked to hypertension [41], we did not find the difference of this ratio between CKD children with normal and abnormal ABPM profile. The reason is possibly because we analyzed gut microbiota in CKD children preceding hypertension onset but not in the stage of established hypertension. According to our data, CKD children with abnormal ABPM group had decreased abundance of genera *Subdoligranulum*, *Faecalibacterium*, *Ruminococcus,* and *Akkermansia*. Low abundance of genera *Subdoligranulum* and *Faecalibacterium* has been identified as a microbial marker in adult hypertensive patients [40,43]. *Ruminococcus* abundance was shown to be deficient in hypertension mice [40]. *Akkermansia* is known as a beneficial gut microbe [44]. Overall, these observations suggest that these certain bacteria populations might have beneficial cardiovascular properties in CKD children to halt the development of hypertension. Moreover, CKD children with abnormal ABPM had decreased abundance of genera *Providencia*, *Gemella*, and *Peptosreptoccocus*. Interestingly, these genera of bacteria have been reported to be involved in TMA production [45]. Thus, whether these microbes play a crucial role on the development of hypertension via mediating the TMA−TMAO metabolic pathway in children with early-stage CKD deserves further clarification. Of note is that a key factor in determining gut microbiota composition is the type of diet in patients with CKD [46]. Since the Mediterranean diet that is rich in fiber has been proven to reduce cardiovascular risk [47], it can be speculated that high-fiber dietary intervention might be an efficient way to control microbiota to further prevent CVD in CKD children [48,49].

This study has several limitations that should be acknowledged. First, a low number of CKD children from one hospital would not be representative of an entire population. Multicenter studies of large numbers of patients may be required to elucidate the true relationship. Second, we presented the associations between certain microbes and BP abnormalities but we do not reveal the pathophysiological mechanism by which those specific microbes contribute to the TMA−TMAO pathway and hypertension. Third, our data might not be applicable to other populations for ethnic reasons. Lastly, there remains a lack of reference for AASI and LVMI to define a cut-off value to indicate normal versus abnormal in a pediatric population. We also did not recruit non-CKD controls because children with CKD stage G1 were served as the controls to compare the differences of BP abnormalities and methylamines levels between two different levels of renal function (i.e., CKD stage G1 vs. stage G2–G4). Although age was different between the two groups, we adjusted the age factor in the multivariate linear regression model.

## 5. Conclusions

Our study in early-stage CKD children demonstrates the associations between gut microbiota-dependent methylamines and BP abnormalities assessed by ABPM. Our results cast a new light on the link between the TMA−TMAO metabolic pathway, gut microbiota, and cardiovascular risk in CKD children. As hypertension and CKD both can originate from early life, early identification of microbial markers related to cardiovascular risk may aid in developing the ideal intervention to improve cardiovascular outcomes in pediatric CKD.

## Figures and Tables

**Table 1:** Descriptive statistics for clinical, anthropometric, and biomedical characteristics of 115 study participants.

Characteristics	
Age, years	11.3 (7.2–15.5)
Male	67 (58.3%)
CKD staging	
Stage G1	79 (68.7%)
Stage G2	27 (23.5%)
Stage G3	7 (6.1%)
Stage G4	2 (1.7%)
CAKUT	76 (66.1%)
Hypertension (by office BP)	49 (42.6%)
Body height, percentile	50 (25–75)
Body weight, percentile	50 (15–85)
Systolic blood pressure, mmHg	112 (101–122)
Diastolic blood pressure, mmHg	71 (66–80)
Body mass index, kg·m^−2^	17.9 (15.2–21.5)
Blood urea nitrogen, mg/dL	13 (10–15)
Creatinine, mg/dL	0.58 (0.46–0.77)
eGFR, mL/min/1.73 m^2^	100.7 (82.3–113.4)
Urine total protein-to-creatinine ratio, mg/g	62.7 (33.9–176.9)
Hemoglobin, g/dL	13.3 (12.7–14.1)
Hematocrit, %	40.5 (38.5–43)
Total cholesterol, mg/dL	169 (144–197)
Low-density lipoprotein, mg/dL	93 (74–118)
Triglyceride, mg/dL	70 (51–92)
Uric acid, mg/dL	5.2 (4.3–6.7)
Sodium, mEq/L	140 (139–141)
Potassium, mEq/L	4.3 (4.2–4.6)
Calcium, mg/dL	9.8 (9.6–10.1)
Phosphate, mg/dL	4.6 (4.2–5)

Data are medians (25th, 75th percentile) or n (%). CAKUT = Congenital anomalies of the kidney and urinary tract.

**Table 2:** Cardiovascular assessments in children and adolescents with chronic kidney disease (CKD) stage G1–G4.

CKD Stage	G1	G2–G4
24-h ABPM	N = 48	N = 27
Awake SBP load (%)	6.5 (0–13)	15 (1–36) *
Asleep SBP load (%)	8.5 (0–25.3)	21 (6–69)
Awake DBP load (%)	2 (0–6)	3 (0–10)
Asleep DBP load (%)	9 (0–18.8)	7 (0–41)
Abnormal ABPM profile (with any of the following abnormalities)	23 (48%)	17 (63%)
Average 24-h BP >95th percentile	2 (4%)	5 (19%)
Average awake BP >95th percentile	2 (4%)	5 (19%)
Average asleep BP >95th percentile	2 (4%)	6 (22%) *
BP load ≥25%	10 (21%)	13 (48%) *
Nocturnal decrease of BP <10%	18 (38%)	13 (48%)
AASI	0.33 (0.21–0.45)	0.41 (0.33–0.57) *
Left ventricular mass (g)	74.6 (54.6–102)	96 (51.8–141.3) *
LVMI (g/m^2.7^)	30.8 (25.2–37.1)	32.7 (28.4–36.3)

Data are medians (25th, 75th percentile) or n (%). * *p* < 0.05 by the Chi-square test or the Mann–Whitney *U*-test. ABPM = 24-h ambulatory blood pressure monitoring. AASI = ambulatory arterial stiffness index. LVMI = left ventricular mass index.

**Table 3:** Plasma and urinary levels of dimethylamine (DMA), trimethylamine (TMA), and trimethylamine N-oxide (TMAO) in children and adolescents with CKD stage G1–G4.

CKD Stage	G1	G2–G4
	N = 73	N = 36
Plasma level, ng/ml		
DMA	91 (81.7–104.8)	124.4 (107.7–147.4) *
TMA	100.8 (83.9–123.1)	112.6 (105.2–138.6) *
TMAO	170.5 (112.9–232.7)	245.7 (129.6–477.1) *
TMAO-to-TMA ratio	1.56 (1.05–2.33)	2.11 (1.17–4.33)
DMA-to-TMAO ratio	0.57 (0.4–0.86)	0.45 (0.34–1)
Urine level, ng/mg Cr		
DMA	222.2 (164.7–281.3)	196.8 (123.8–243.2) *
TMA	3.18 (2.46–5.75)	2.96 (1.44–6.62)
TMAO	271.1 (167.3–417.8)	183.8 (107.5–291.6) *
TMAO-to-TMA ratio	87.5 (55.8–113.7)	68.7 (38.2–112.9)
DMA-to-TMAO ratio	0.84 (0.55–1.15)	0.89 (0.51–1.48)
Fractional excretion of DMA	44.2 (30.2–61.2)	48.8 (36.9–69.6)
Fractional excretion of TMA	1.6 (1.13–2.84)	1.87 (1.13–4.99)
Fractional excretion of TMAO	82.3 67.9–96.8)	81.3 (61–92.1)

Data are medians (25th, 75th percentile). * *p* < 0.05 by the Mann–Whitney *U*-test. DMA = dimethylamine; TMA = trimethylamine; TMAO = trimethylamine N-oxide.

**Table 4:** Correlation between plasma and urinary methylamines and cardiovascular risk factors in children with CKD stage G1–G4.

CV Risk Factors	Awake SBP Load		Asleep SBP Load		Awake DBP Load		Asleep DBP Load		LV Mass	
	*r*	*p*	*r*	*p*	*r*	*p*	*r*	*p*	*r*	*p*
Plasma										
DMA	0.144	0.221	0.141	0.232	0.117	0.32	0.039	0.74	0.059	0.534
TMA	0.115	0.329	0.133	0.26	−0.029	0.807	0.137	0.244	0.093	0.326
TMAO	0.113	0.339	−0.018	0.879	0.088	0.457	−0.08	0.945	0.024	0.797
Urine										
DMA	−0.235	0.043 *	−0.289	0.012 *	−0.288	0.012 *	−0.022	0.854	−0.554	<0.001 *
TMA	0.212	0.067	0.07	0.548	0.105	0.368	0.131	0.261	−0.226	0.016 *
TMAO	0.043	0.716	−0.223	0.055	0.013	0.91	0.003	0.980	−0.324	<0.001 *

*p < 0.05 by Spearman’s correlation coefficient. DMA = dimethylamine; TMA = trimethylamine; TMAO = trimethylamine N-oxide.

**Table 5:** Adjusted regression model estimates of the association between plasma and urinary methylamines and cardiovascular risk factors in children with CKD stage G1–G4.

Dependent Variable	Explanatory Variable	Adjusted ^a^		Model	
		*Beta*	*p* Value	*r*	*p* Value
Office DBP	Urine TMA	−0.227	0.009	0.479	<0.001
	Plasma TMA	0.202	0.02		
	Urine DMA-to-TMAO ratio	−0.221	0.012	0.441	<0.001
Awake DBP	Plasma TMA	0.283	0.015	0.283	0.015
Asleep DBP	Plasma TMA	0.232	0.036	0.423	0.001
LV mass	Urine DMA-to-TMAO ratio	−0.148	0.018	0.771	<0.001
LVMI	Plasma DMA-to-TMAO ratio	−0.218	0.012	0.465	<0.001

DMA = dimethylamine; TMA = trimethylamine; TMAO = trimethylamine N-oxide. LVMI = left ventricular mass index. ^a^ Adjusted for age, sex, eGFR, and other methylamines.
